# Storage-dependent variability in Alzheimer disease-related plasma biomarker results using the Fujirebio Lumipulse G1200 platform

**DOI:** 10.1093/jnen/nlaf115

**Published:** 2025-10-10

**Authors:** Ibrahim Choucair, Tiffany L Lee, Linda J Van Eldik, Jeffrey L Dage, Brian T Gold, Erin L Abner, Gregory A Jicha, Peter T Nelson

**Affiliations:** Department of Pathology and Laboratory Medicine, University of Kentucky, Lexington, KY, United States; Sanders-Brown Center on Aging, University of Kentucky, Lexington, KY, United States; Sanders-Brown Center on Aging, University of Kentucky, Lexington, KY, United States; Sanders-Brown Center on Aging, University of Kentucky, Lexington, KY, United States; Department of Neuroscience, University of Kentucky, Lexington, KY, United States; Department of Neurology, Indiana University, Indianapolis, IN, United States; Stark Neurosciences Research Institute, Indiana University, Indianapolis, IN, United States; Sanders-Brown Center on Aging, University of Kentucky, Lexington, KY, United States; Department of Neuroscience, University of Kentucky, Lexington, KY, United States; Sanders-Brown Center on Aging, University of Kentucky, Lexington, KY, United States; Department of Epidemiology & Environmental Health, University of Kentucky, Lexington, KY, United States; Sanders-Brown Center on Aging, University of Kentucky, Lexington, KY, United States; Department of Neurology, University of Kentucky, Lexington, KY, United States; Department of Pathology and Laboratory Medicine, University of Kentucky, Lexington, KY, United States; Sanders-Brown Center on Aging, University of Kentucky, Lexington, KY, United States

**Keywords:** Alzheimer disease, amyloid-beta (Aβ40, Aβ42), glial fibrillary acidic protein (GFAP), neurofilament light (NfL), phosphorylated tau (pTau181, pTau217), plasma biomarker, pre-analytical stability

## Abstract

Alzheimer disease plasma biomarkers have emerged as minimally invasive, cost-effective tools for early diagnosis and disease monitoring yet their stability under common “real world” pre-analytical conditions remains incompletely characterized. We evaluated the stability of six plasma biomarkers, Aβ40, Aβ42, pTau181, pTau217, NfL, and glial fibrillary acidic protein (GFAP) using the Fujirebio Lumipulse G1200 platform. Plasma samples were initially collected from four healthy and cognitively unimpaired volunteers. Samples were stored under four conditions: room temperature (0-4 h), +4 °C (1-10 days), −20 °C (1-3 weeks), and −80 °C (4-8 weeks). In this pilot study, Aβ40 and Aβ42 remained generally stable. In contrast, pTau181 readings exhibited marked elevations in frozen samples, while pTau217 showed modest early fluctuations followed by significant decreases with prolonged storage. Next, we recruited 12 additional participants (six cognitively normal and six with mild cognitive impairment [MCI]), and their plasma samples were analyzed both fresh and after 4 weeks of storage at −80 °C. Among these participants, pTau181 readouts were significantly higher, and pTau217 were lower, in −80 °C frozen in comparison to never-frozen samples. These findings underscore the critical need for biomarker-specific sample workup and handling protocols and indicate that results for fresh plasma cannot be assumed to be the same as for frozen samples.

## INTRODUCTION

Alzheimer disease (AD) is a progressive neurodegenerative disorder that is the most common cause of dementia.^1^ It is pathologically characterized by the accumulation of extracellular amyloid-β (Aβ) plaques and intracellular neurofibrillary tangles composed of hyperphosphorylated tau.^2^ Early diagnosis and intervention are crucial for effective management and can potentially slow disease progression.^3^ As such, there has been a growing emphasis on the development and validation of fluid biomarkers to aid in diagnosis of AD, including Aβ42, Aβ42/40, NfL, glial fibrillary acidic protein (GFAP), total tau (t-tau), and phosphorylated tau (p-tau).^4–6^

The measurement of plasma biomarkers for AD and related disorders (ADRD) has gained significant traction as a relatively non-invasive and cost-effective but still molecular-specific alternative to more invasive cerebrospinal fluid (CSF) biomarkers and positron emission tomography (PET) imaging.^5^ Plasma biomarkers offer promising utility in clinical and research settings, with important implications to clinical practice as we embark on the era of disease-modifying drugs that are used to prevent or reverse pathological hallmarks of ADRD.^7^^,^^8^ Among the most widely studied biomarkers are Aβ peptides (Aβ40 and Aβ42), phosphorylated tau (pTau181 and pTau217), neurofilament light chain (NfL), and GFAP, which are useful analytes for aiding in early diagnosis and understanding disease progression.^4^^,^^5^^,^^9^

Despite their potential, the stability of ADRD plasma biomarkers under common “real world” pre-analytical conditions remains incompletely characterized. Factors such as sample storage temperature, duration of storage before processing, and freeze-thaw cycles can significantly impact biomarker integrity, potentially leading to variability and spurious results.^10^ Thus, rigorous stability studies are essential to establish optimal, standardized handling protocols, ensuring the reliability and reproducibility of biomarker measurements. While prior investigations have addressed the pre-analytical variability of some ADRD biomarkers, few previous published studies systematically evaluated ADRD plasma biomarker stability under common practices in laboratory medicine testing, including variations in room temperature, refrigeration, and freezing at −20 °C and −80 °C.^11^^,^^12^

This study aimed to address this gap by investigating the stability of Aβ40, Aβ42, pTau181, pTau217, NfL, and GFAP under clinically relevant conditions over extended periods. The findings provide critical insights into optimal sample management practices, enhancing the reliability of plasma biomarker assays for ADRD research and diagnostics.

## METHODS

### Sample collection and handling

**Figure 1.**
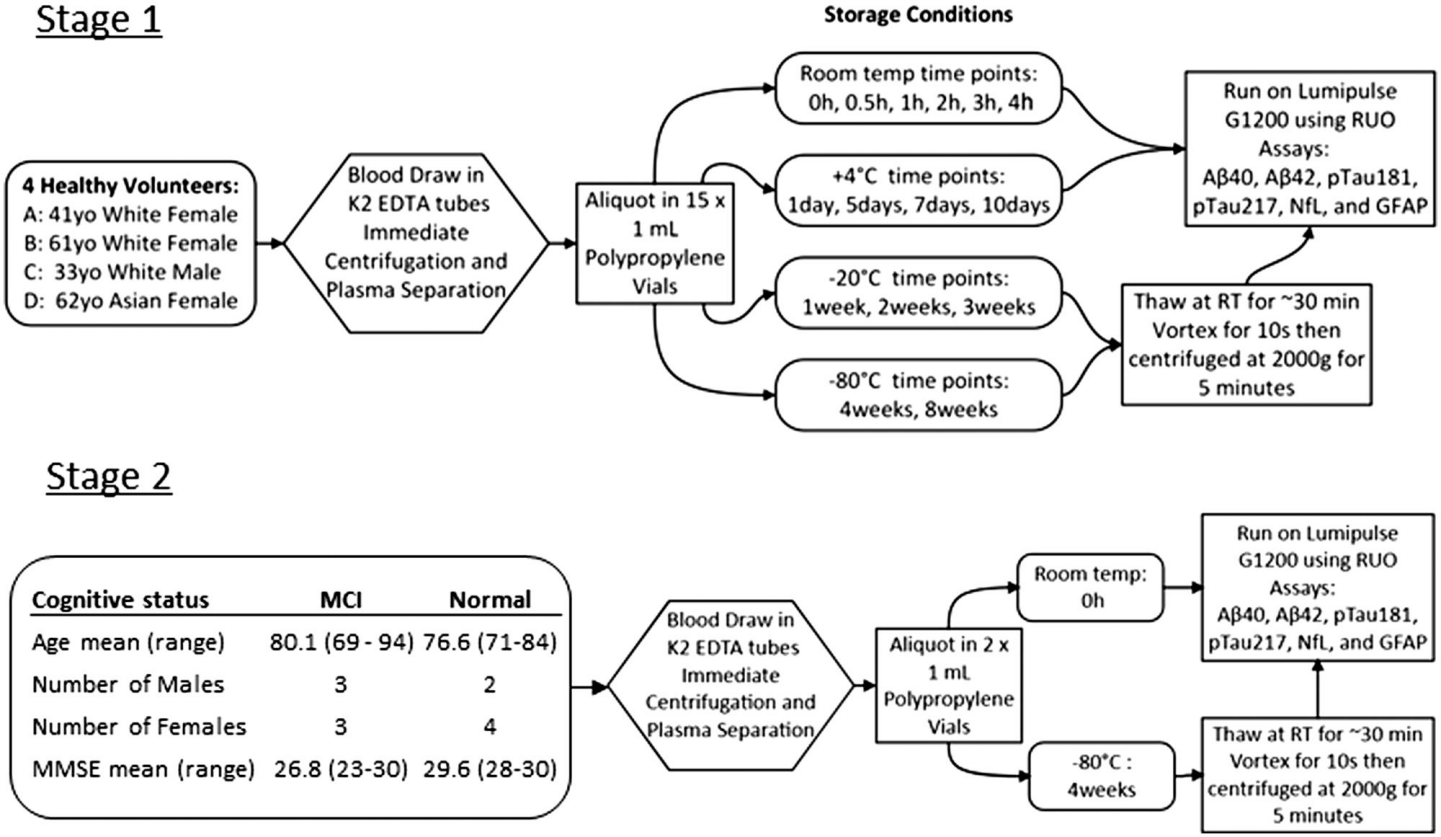
Study design, participant demographics, and sample workflow for plasma biomarker stability evaluation. Mild cognitive impairment (MCI), Mini-Mental State Examination (MMSE).

Blood was initially collected from four healthy volunteers (hereafter referred to as Stage 1; comprising participants A, B, C, and D), who provided informed consent for research use of their samples. The blood was drawn into three 10 mL K2 EDTA tubes (BD 366643), inverted 5-10 times, and immediately centrifuged in less than 10 min. Plasma was aliquoted into 15 separate 1 mL portions in polypropylene screw-cap vials, then stored under four temperature conditions: room temperature (*n* = 6 aliquots), +4 °C (*n* = 4), −20 °C (*n* = 3), and −80 °C (*n* = 2). Room temperature samples were analyzed at 0 (within 5 min of the centrifugation), 0.5, 1, 2, 3, and 4 h. Samples stored at +4 °C were analyzed at 1, 5/7, and 10 days; those at −20 °C were analyzed at 1, 2, and 3 weeks; and those at −80 °C were analyzed at 4 and 8 weeks. At room temperature, the 0.5-h sample for participant C was excluded from analysis due to the presence of red blood cell contamination. Samples from participants A and B were processed together, as was the case for participants C and D; consequently, only samples from A and B were analyzed at the 5-day time point, while samples from C and D were analyzed at the 7-day time point. Due to a shortage of reagents, the samples from C and D stored at −20 °C for 8 weeks were not analyzed. Finally, GFAP testing was introduced in our laboratory after samples from A and B had already been analyzed; therefore, its stability was assessed only for C and D. Next, we recruited 12 additional participants (Stage 2 comprising five males and seven females; six of these participants were cognitively normal and six with mild cognitive impairment [MCI]), and their samples were analyzed both fresh and after 4 weeks of storage at −80 °C. The study design, participant demographics, and sample workflow used for evaluating plasma biomarker stability are illustrated in Figure 1.

### Plasma biomarkers measurement

Aβ40, Aβ42, pTau181, pTau217, NfL, and GFAP were measured in plasma using a research-use-only (RUO) method on the Lumipulse G1200 platform, following the instructions provided in the manufacturer’s kit insert. The analytical measurement ranges for each biomarker were: Aβ40 (0.1-5000 pg/mL), Aβ42 (0.1-1000 pg/mL), pTau181 (0.05-60 pg/mL), pTau217 (0.03-10 pg/mL), NfL (2-5000 pg/mL), and GFAP (2-5000 pg/mL). Frozen samples were thawed at room temperature and inspected visually until completely thawed. Once thawed, samples were left for at least 30 min to equilibrate to room temperature prior to testing, vortexed for 10 seconds, centrifuged at 2000×g for 5 min, and supernatants analyzed. Samples stored at +4 °C were brought to room temperature on the bench for at least 30 min before testing. Testing was performed immediately after preparation. For every batch, both low- and high-level quality control (QC) samples were run, and overall assay performance for all analytes is monitored biannually through peer comparison via the Alzheimer’s Association QC program.^13^

### Data analysis

Biomarker concentrations under each experimental condition were normalized to the room temperature 0‐h values and expressed as percent deviations from baseline. Group mean percent deviations were then calculated for each sample and condition. The acceptance criterion for all plasma biomarkers was set at ±20% deviation from reference sample, considering that the 0-h (reference sample) has an inherent error on its own. For reference, the percent total error calculated from quality control samples analyzed over the study period for all analytes is as follows: Aβ40 17.7%, Aβ42 16.8%, pTau181 13.7%, pTau217 11.0%, NfL 26.0%, and GFAP 27.0%. Results were plotted to visualize changes over time. Generalized estimating equations (GEE) on linear regression with robust standard errors were used to test the effects of storage temperature on mean biomarker levels over time in the Stage 1 study using the SAS 9.4. For this analysis, because time in storage and temperature were confounded (ie, each time point occurred within a single temperature condition), the last time point in each temperature condition was selected (ie 4 h, 10 days, and 3 weeks), except for the −80 °C condition where the first time point (4 weeks) was used due to missing data at the last (8 weeks) time point. The Wilcoxon test using GraphPad Prism 10 was used to assess the difference between the 12 samples analyzed fresh and after storage at −80 °C. A *P* value less than .05 was considered statistically significant.

## RESULTS

In Stage 1, EDTA plasma samples from 4 participants (three females and one male, aged 33 to 62 years) were analyzed across all described storage conditions using the Fujirebio Lumipulse system. In Stage 2, samples from 12 participants (five males and seven females; six cognitively normal and six with MCI; aged 69-94 years) were analyzed fresh and after storage at −80 °C only. Demographic details are presented in Figure 1.

### Stage 1

#### Aβ markers (Aβ40 and Aβ42)

**Figure 2.**
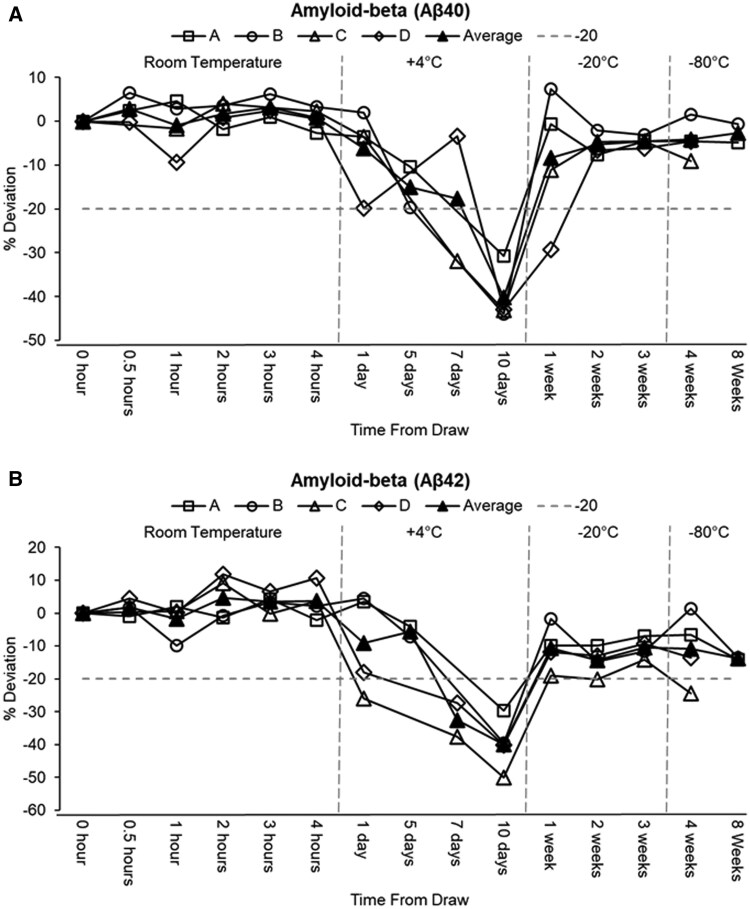
Stability of plasma amyloid-β biomarkers (Aβ40 [panel A] and Aβ42 [panel B]) under varying storage conditions. Percent (%) deviation of all samples is normalized to the 0-h time point at room temperature. Each mark on the graph is an aliquot taken fresh and stored at the specified temperature and time conditions.

Analysis of the plasma proteins stored at room temperature revealed that the amyloid‐β peptides were relatively stable over the 4‐h period (Supplementary Table 1; Figure 2A and B). Both proteins exhibited minor fluctuations, staying within a 5% deviation from baseline. When stored at 4°C, both Aβ40 and Aβ42 exhibited progressive declines with extended storage. The average Aβ40 concentration decreased from 254.79 pg/mL at 1 day to 163.63 pg/mL at 10 days (−40%) with stability within 20% deviation up to 7 days. Aβ42 declined by up to −40% by day 10, with stability maintained within ±20% for the first 5 days. This was further supported by the GEE models, which showed that compared to fresh samples, Aβ40 and Aβ42 levels were significantly reduced at 4 °C (−109.0 pg/mL, *P* < .0001) and (−11.4 pg/mL, *P* < .0001) respectively. Interestingly, the Aβ42/Aβ40 ratio remained within ±20% after 10 days. The GEE model showed significant deviation for both Aβ40 and Aβ42 at −20 °C and −80 °C. Aβ40 levels were significantly reduced at −20 °C (−12.7 pg/mL, *P* < .0001), and −80 °C (−10.9 pg/mL, *P* = .0192). Aβ42 exhibited similar patterns, with significantly lower values at −20 °C (−3.0 pg/mL, *P* < .0001), and −80 °C (−3.0 pg/mL, *P* = .0132). These deviations, while statistically significant, stayed within acceptable limits. Under −20 °C storage conditions, the Aβ peptides remained comparatively stable; Aβ40 showed modest increases from a baseline decline of −8% at 1 week to −5% at 3 weeks, while Aβ42 maintained mean reductions of approximately 10 to 13% over the same period. After 4 and 8 weeks at −80 °C, the Aβ peptides remained robust. Aβ40 deviated by only −4% and −3% at 4 and 8 weeks, respectively, while Aβ42 declined moderately by approximately 10 to14%.

#### Tau markers (pTau181 and pTau217)

**Figure 3.**
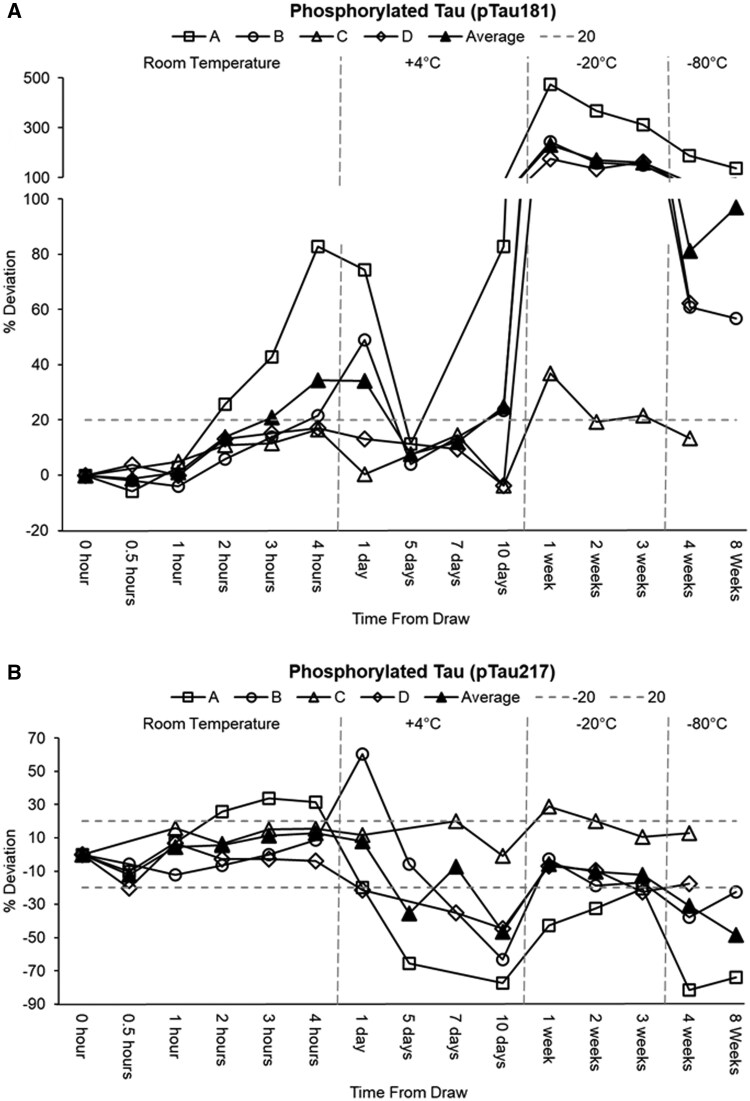
Stability of plasma tau biomarkers (pTau181 [panel A] and pTau217 [panel B]) under varying storage conditions. Percent (%) deviation of all samples is normalized to the 0-h time point at room temperature. Each mark on the graph is an aliquot taken fresh and stored at the specified temperature and time conditions.

Tau levels demonstrated time‐dependent shifts (Supplementary Table 1; Figure 3A and B). Plasma pTau181 increased progressively when left at room temperature from an average baseline of 1.00 pg/mL, with deviations reaching +14% at 2 h and +34% at 4 h. A similar but less pronounced pattern was observed for pTau217 at room temperature, which showed an initial decline (−12% at 0.5 h) followed by a gradual increase, culminating in a + 13% deviation at 4 h, which is well within the 20% analytical variability. These findings were validated by GEE model results, which showed pTau181 and pTau217 levels were significantly higher at room temperature (+0.230 pg/mL, *P* = .001) and (+0.031 pg/mL, *P* = .045) respectively. When stored at 4 °C (1-10 days), pTau181 displayed a biphasic pattern mostly driven by sample A, with an elevated mean of 1.15 pg/mL at 1 day (+34%) and 1.07 pg/mL at 10 days (+25%), despite a transient acceptable stability at 5 and 7 days. pTau217 levels were notably reduced after prolonged storage, with average declines of −35% and −46% at 5 and 10 days, respectively. For pTau217, the GEE model identified a statistically significant decrease at 4 °C (−0.075 pg/mL, *P* = .026) with no significant change for pTau181 at 4 °C (*P* = .33). Under −20 °C conditions (1-3 weeks), pTau181 exhibited marked increases relative to baseline, with average concentrations rising by 232% at 1 week and remaining elevated (161% above baseline) at 3 weeks with an estimated difference by the GEE model of +0.818 pg/mL, *P* < .0001. In contrast, pTau217 was relatively stable, showing only slight decreases (−6% to −12%) with no significant decrease in the GEE model. At −80 °C (4-8 weeks), pTau181 remained elevated relative to baseline, with average increases of 81% at 4 weeks and 97% at 8 weeks (noting that the 8‐week data were derived from partial datasets) with an estimated difference of +0.413 pg/mL, *P* < .0001 at 4 weeks in the GEE model. Conversely, pTau217 experienced substantial decreases (−31% at 4 weeks and −48% at 8 weeks) but still with no significant change in the GEE model.

#### NfL and GFAP

**Figure 4.**
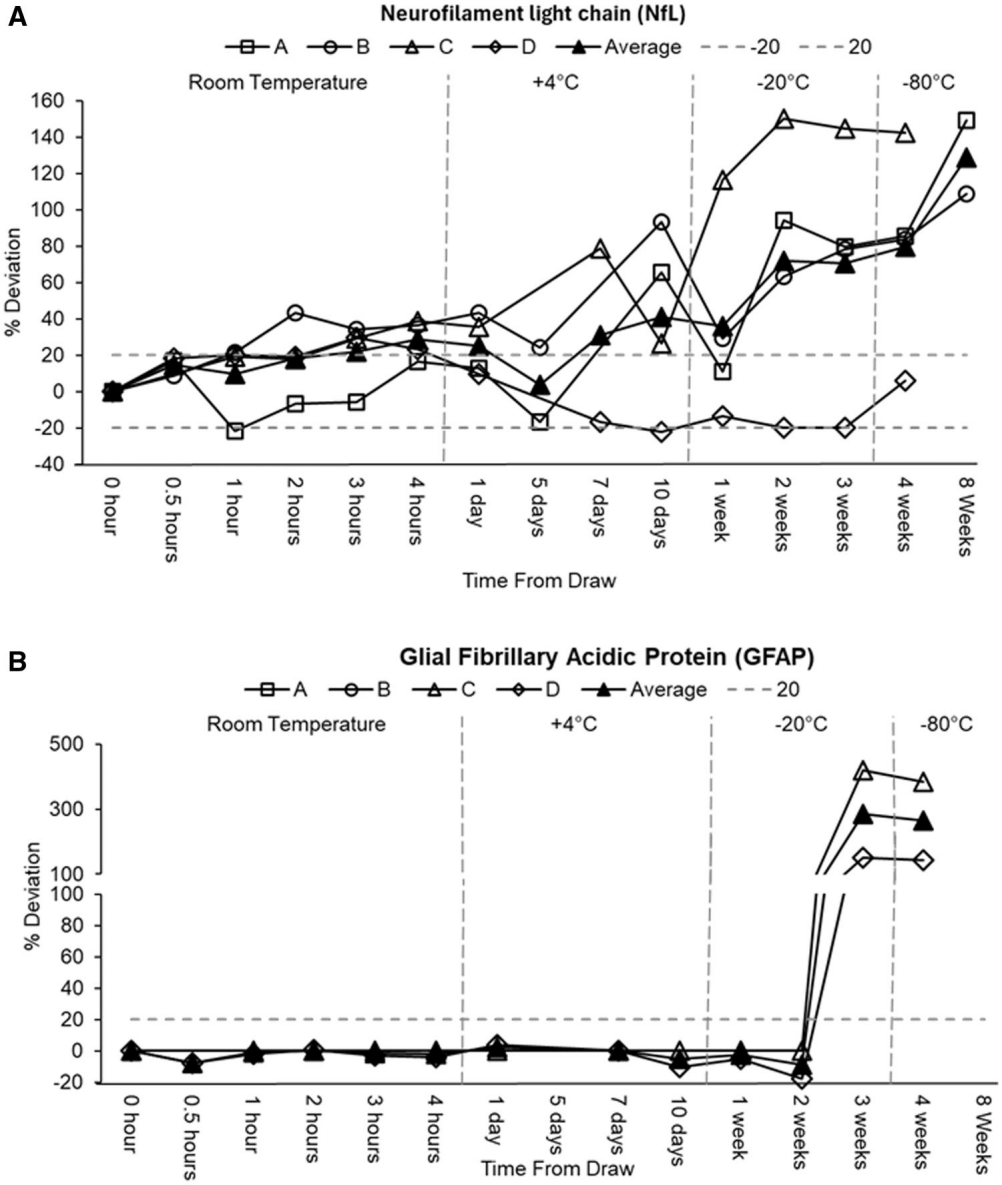
Stability of plasma neurofilament light chain (NfL; panel A) and glial fibrillary acidic protein (GFAP; panel B) under varying storage conditions. Percent (%) deviation of all samples is normalized to the 0-h time point at room temperature. Each mark on the graph is an aliquot taken fresh and stored at the specified temperature and time conditions.

Neurofilament light (NfL) levels increased consistently across storage conditions (Supplementary Table 1; Figure 4A). At room temperature, the average NfL increased from 6.44 pg/mL at baseline to 8.23 pg/mL at 4 h (a 29% deviation), with the deviation exceeding 20% (22%) by 3 h with an estimated difference by the GEE model of +1.80 pg/mL, *P* < .0001. Under 4 °C storage (1-10 days), NfL demonstrated variability, with average increases from 7.85 pg/mL at 1 day (+25%) to 8.00 pg/mL at 10 days (+41%). At −20 °C (1-3 weeks), NfL levels were significantly elevated, with average increases ranging from +36% at 1 week to +71% at 3 weeks. Under −80 °C storage (4-8 weeks), NfL concentrations rose markedly by 79% at 4 weeks and 129% at 8 weeks. The GEE analysis further supported these trends, showing a non-significant increase in NfL levels at −20 °C (+2.9 pg/mL, *P* = .078) and a significant increase at −80 °C (+3.77 pg/mL, *P* = .0003).

GFAP exhibited variable behavior (Supplementary Table 1; Figure 4B). At room temperature, GFAP remained largely unaltered in participant C (constant at 4.0 pg/mL) and showed only modest fluctuations in participant D (ranging from −8% to −2%). During 4 °C storage, GFAP remained stable in the two participants with available data, with average changes of less than 6% over the 10‐day period. However, under −20 °C conditions, GFAP data were highly variable; while initial values were near baseline at 1 and 2 weeks, a dramatic increase of +286% was observed at 3 weeks. At −80 °C, with available data only at 4 weeks, GFAP demonstrated a substantial increase of 264% above baseline. GFAP was not included in the GEE models due to missing data for participants A and B.

### Stage 2

**Figure 5.**
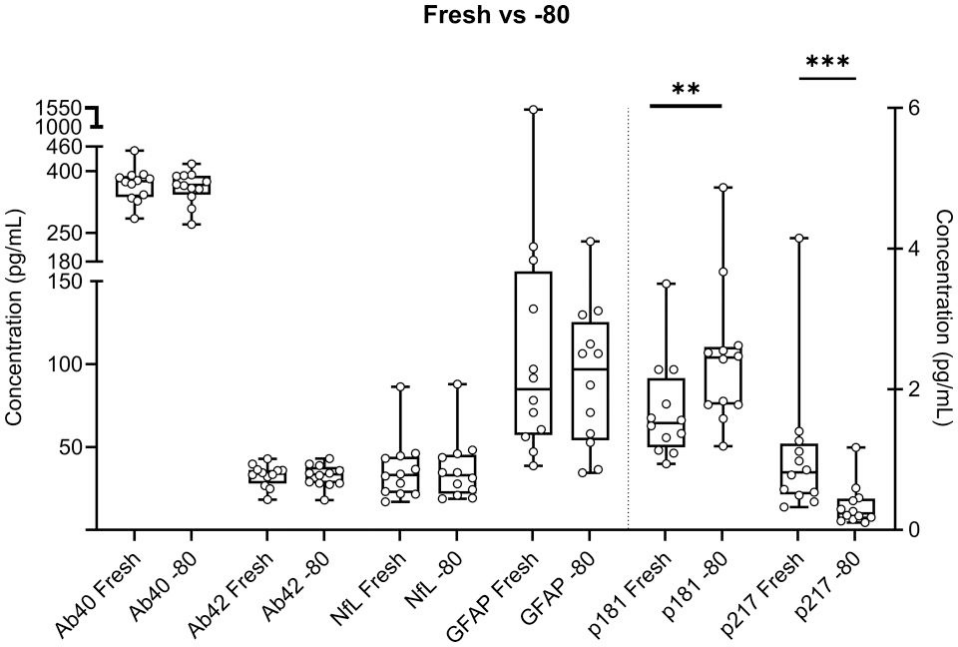
Plasma biomarkers for AD and related disorders measured Fresh vs −80 °C. Samples were analyzed fresh and after 4 weeks at −80 °C. Each marker represents an individual sample; whiskers represent the minimum and maximum values. The Wilcoxon test was used to assess the difference between the 12 samples analyzed fresh and after storage at −80 °C and a *P* value less than .05 was considered statistically significant. **P = .0024; ***P = .0005.

The stability of the amyloid‐β peptides seen in stage 1 was confirmed in Stage 2 comparing fresh to stored samples at −80 °C for four weeks, with *P* values of .47 and .73 for Aβ40 and Aβ42, respectively. Likewise, both Tau markers demonstrated a similar but less pronounced trend in Stage 2 of the study, with statistically significant *P* values of .002 for pTau181 and .0005 for pTau217. NfL demonstrated high stability when comparing fresh versus −80 °C storage with a *P* value of .67, which contradicts the earlier results. Notably, in Stage 1 three out of four samples had low concentrations (3.8, 4.5, and 5.5 pg/mL), while sample D, which exhibited similar stability to Stage 2, had a higher concentration of 11.8 pg/mL. In contrast, the average NfL concentration in Stage 2 of the study was 36.1 pg/mL. GFAP exhibited a similar but less pronounced increase in samples stored at −80 °C compared to fresh samples with a non-significant *P* value of .34. Notably, GFAP levels were substantially higher in Stage 2 cohort, averaging 85.7 pg/mL (after removing two extreme outliers), versus 4.0 pg/mL and 15.3 pg/mL in Stage 1 samples C and D. Excluding the two outliers with fresh concentrations of 216.2 pg/mL and 1504.1 pg/mL yields a significant *P* value of .0098 (Supplementary Table 2; Figure 5).

## DISCUSSION

Recent work suggested high clinical value of plasma biomarkers, such as Aβ40, Aβ42, phosphorylated tau isoforms (pTau181, pTau217), NfL, and GFAP, in diagnosing and staging AD and other neurodegenerative conditions.^4^^,^^9^ As these markers are ushering a new era in AD/ADRD diagnosis, it is important to understand the pre-analytical stability of each of them. While others have published studies discussing varying storage stability of these analytes,^12^^,^^14–18^ we designed a study that incorporated “real world” clinical sample handling, and differences between research (usually involving frozen/batched samples) and clinical laboratory (typically never-freezing) storage conditions. Our study demonstrates that pre‐analytical storage conditions exert a substantial influence on plasma biomarker concentrations relevant to neurodegenerative diseases. To our knowledge, this is the first study that tests the −80 °C storage condition against a never-frozen or refrigerated sample, centrifuged immediately after the blood draw, aliquoted, and analyzed within 30 min of blood draw, as the patient specific reference and benchmark.

While pre-analytical stability of Aβ proteins in CSF has been a major concern (especially with regard to the plastic construction of the collection tube [10, 15]), our study demonstrated that plasma samples stored in polypropylene screw-cap tubes maintained overall good stability under the evaluated storage conditions. More specifically, our findings demonstrated that plasma Aβ40 and Aβ42 exhibit minimal changes during short‐term (0-4 h) storage at room temperature. This stability was consistent with previous work showing that, when processed promptly, Aβ peptides are relatively robust to pre‐analytical variability.^17^^,^^19^ Our data showed that Aβ peptides are stable at 4 °C up to 5 days. However, the marked decline of detected peptide observed during extended storage at 4 °C, up to approximately 40% reduction over 10 days, confirmed earlier reports that showed Aβ peptides in refrigerated samples are stable when tested up to 3 days but not stable when tested at 8 to 9 days storage.^12^^,^^14^^,^^19^ Interestingly, our data showed that this decline is diminished when using the Aβ42/Aβ40 ratio, likely because they showed a very similar pattern of detected peptide levels, both showing a steep decline at 10 days (Fig. 1), which cancels out when using their ratio. In contrast, the comparatively minor deviations under −20 °C and especially −80 °C storage conditions supported recommendations for ultra‐low temperature storage to preserve sample integrity for long‐term biobanking.^20^ These observations suggest that while short‐term handling does not compromise Aβ measurements, prolonged storage at RT or 4 °C may yield concentrations that are artificially low, potentially affecting clinical interpretations.

In the present study, tau‐related markers displayed different time‐dependent changes. Detected plasma pTau181 levels increased notably even under room temperature conditions, with deviations reaching up to 34% over 4 h, and became markedly elevated under both −20 and −80 °C storage. This sensitivity to storage conditions aligned with recent findings that compared −80 °C and/or −20 °C with 4 °C storage conditions.^12^^,^^14^ It also indicates that prior studies that used −80 °C storage to “lock in” experimental condition and batch all samples together^11^^,^^18^ may have been based on suboptimal assumptions of stability. One study showed excellent stability for pTau181 at room temperature up to 24 h storage as well as after 4X freeze/thaw cycles at −20 and −80 °C (time was not defined) using the Roche system.^16^ We hypothesize that several factors may have contributed to this apparent discrepancy. First, variations in the analytical systems could be influential. Second, differences in study design, particularly with respect to time as we demonstrated to be major determinants of sample stability. Finally, we observed that pTau181 concentrations, especially in samples with lower absolute levels, exhibited markedly higher percent deviations. Our raw data is shown in Supplementary Tables 1 and 2. In contrast to pTau181, pTau217 exhibited overall better robustness to the storage conditions tested but showed some reductions during extended storage at 4 and −80 °C as replicated in both stages of the current study. Notably, samples with higher absolute pTau217 levels exhibited markedly greater percent deviations. The pTau217/Aβ42 ratio also differed significantly between fresh and −80 °C samples (*P* = .0005), indicating that further investigation of this important AD/ADRD analyte is warranted. As far as we are aware, this is the first study that investigated extended stability for pTau217, but our results are in line with the Roche study that showed stability at room temperature and 4 °C up to 24 h.^16^

Detected levels of NfL increased (in comparison to our benchmark fresh sample results), consistently across storage conditions. At room temperature, NfL levels rose by nearly 29% within 4 h, and even more dramatic elevations were observed with extended storage at 4, −20, and −80 °C. The 12-participant study did not replicate these findings, showing no statistically significant difference between fresh and −80 °C frozen samples. This discrepancy may be attributable to the absolute NfL concentrations in those samples. This was the first study that tested extended storage on various conditions and compared the results, for benchmarking, to the fresh non-delayed tested sample. Again, our findings with NfL contradicted the studies that used −80 °C storage to lock-in experimental condition and batch all samples together^11^^,^^18^^,^^21^ and with the Roche study showing NfL stability at room temperature for 24 h.^16^ In contrast to NfL, detected GFAP levels exhibited relatively minor fluctuations at room temperature and during 4 °C storage, suggesting stability under these conditions consistent with previous reports that tested up to two weeks at −20 °C.^11^^,^^18^ However, the marked increases in detected GFAP levels observed at −20 and −80 °C at 3, 4, and 8 weeks indicated that this biomarker may be particularly sensitive to extended storage at freezing temperatures.

The inclusion of non-frozen samples is a considerable strength of the current study, which should raise awareness in the field as we move toward more biomarker analyses in the clinical (usually non-freezing) context. However, there are several limitations to the current study, that may partially influence the interpretation of results. Blood samples across all storage conditions were obtained from a limited number of participants, especially in Stage 1, but the trend between Stages 1 and 2 showed that there was some overlap and statistical significance in Stage 2. More analyses across human participants with varying levels of ADRD risk and clinical expression of disease are required in future studies. Another limitation of the current study is that some participant samples were missing specific temperature data-points restricting the ability to fully delineate time-dependent effects. Additionally, we only measured −80 °C storage up to eight weeks, which may not capture the longer-term effects of sample storage for several months or years, as is common in biobank practice. Despite these constraints, our results clearly highlight the urgent need for standardized pre-analytical protocols tailored to each biomarker’s unique stability profile. Additionally, these results stress the importance of validating storage conditions during assay development, with clear guidelines on acceptable bias thresholds under different conditions.

In summary, this study highlights the possible impacts of storage conditions on the stability of key ADRD plasma biomarkers, when using the Fujirebio Lumipulse system. These findings underscore that preanalytical sample handling can influence the analytic results, particularly for sensitive biomarkers like pTau181 and GFAP. The implications of these results are significant for clinical and research applications, particularly multi-center studies and biobanking efforts. Differences in storage protocols can introduce substantial variability, potentially confounding clinical interpretations and reducing reproducibility across sites. Since refrigeration/freezing may be impactful, the results of a clinical study (often not frozen) may differ appreciably from a research study, where samples tend to be frozen and “batched” for future analyses.

## Supplementary Material

nlaf115_Supplementary_Data

## References

[1] 2024 Alzheimer’s disease facts and figures. Alzheimers Dement. 2024;20:3708-3821. 38689398 10.1002/alz.13809 PMC11095490

[2] Montine TJ, , Phelps CH, , Beach TG, et al National institute on aging-alzheimer's association guidelines for the neuropathologic assessment of Alzheimer's disease: a practical approach. Acta Neuropathol. 2012;123:1-11. 22101365 10.1007/s00401-011-0910-3 PMC3268003

[3] Jicha GA , Abner EL, Coskun EP, et al Perspectives on the clinical use of anti-amyloid therapy for the treatment of Alzheimer’s disease: insights from the fields of cancer, rheumatology, and neurology. Alzheimers Dement (N Y). 2024;10:e12500. 39296920 10.1002/trc2.12500 PMC11409193

[4] Rajendran K , Krishnan UM. Biomarkers in Alzheimer’s disease. Clin Chim Acta. 2024;562:119857. 38986861 10.1016/j.cca.2024.119857

[5] An C , Cai H, Ren Z, et al Biofluid biomarkers for Alzheimer’s disease: past, present, and future. Med Rev (2021). 2024;4:467-491. 39664082 10.1515/mr-2023-0071 PMC11629312

[6] Hansson O , Blennow K, Zetterberg H, et al Blood biomarkers for Alzheimer’s disease in clinical practice and trials. Nat Aging. 2023;3:506-519. 37202517 10.1038/s43587-023-00403-3 PMC10979350

[7] Pais MV , Forlenza OV, Diniz BS. Plasma biomarkers of Alzheimer’s disease: a review of available assays, recent developments, and implications for clinical practice. J Alzheimers Dis Rep. 2023;7:355-380. 37220625 10.3233/ADR-230029 PMC10200198

[8] Howe MD , Britton KJ, Joyce HE, et al Clinical application of plasma P-tau217 to assess eligibility for amyloid-lowering immunotherapy in memory clinic patients with early Alzheimer’s disease. Alzheimers Res Ther. 2024;16:154. 38971815 10.1186/s13195-024-01521-9 PMC11227160

[9] Arslan B , Zetterberg H, Ashton NJ. Blood-based biomarkers in Alzheimer’s disease—moving towards a new era of diagnostics. Clin Chem Lab Med. 2024;62:1063-1069. 38253262 10.1515/cclm-2023-1434

[10] Vanderstichele H , Bibl M, Engelborghs S, et al Standardization of preanalytical aspects of cerebrospinal fluid biomarker testing for Alzheimer’s disease diagnosis: a consensus paper from the Alzheimer’s biomarkers standardization initiative. Alzheimers Dement. 2012;8:65-73. 22047631 10.1016/j.jalz.2011.07.004

[11] Verberk IMW , Misdorp EO, Koelewijn J, et al Characterization of pre-analytical sample handling effects on a panel of Alzheimer’s disease-related blood-based biomarkers: results from the standardization of Alzheimer’s blood biomarkers (SABB) working group. Alzheimers Dement. 2022;18:1484-1497. 34845818 10.1002/alz.12510 PMC9148379

[12] Musso G , Cosma C, Zaninotto M, et al Pre-analytical variability of the lumipulse immunoassay for plasma biomarkers of Alzheimer’s disease. Clin Chem Lab Med. 2023;61:e53-e56. 36423341 10.1515/cclm-2022-0770

[13] The Alzheimer’s Association QC program for CSF and blood biomarkers. https://www.gu.se/en/neuroscience-physiology/the-alzheimers-association-qc-program-for-csf-and-blood-biomarkers

[14] Mansilla A , Canyelles M, Ferrer R, et al Effects of storage conditions on the stability of blood-based markers for the diagnosis of Alzheimer’s disease. Clin Chem Lab Med. 2023;61:1580-1589. 37083158 10.1515/cclm-2023-0245

[15] Lewczuk P , Beck G, Esselmann H, et al Effect of sample collection tubes on cerebrospinal fluid concentrations of tau proteins and amyloid beta peptides. Clin Chem. 2006;52:332-334. 16449222 10.1373/clinchem.2005.058776

[16] Kurz C , Stockl L, Schrurs I, et al Impact of pre-analytical sample handling factors on plasma biomarkers of Alzheimer’s disease. J Neurochem. 2023;165:95-105. 36625424 10.1111/jnc.15757

[17] Rozga M , Bittner T, Batrla R, et al Preanalytical sample handling recommendations for Alzheimer’s disease plasma biomarkers. Alzheimers Dement (Amst). 2019;11:291-300. 30984815 10.1016/j.dadm.2019.02.002 PMC6446057

[18] Sunde AL , Alsnes IV, Aarsland D, et al Preanalytical stability of plasma biomarkers for Alzheimer’s disease pathology. Alzheimers Dement (Amst). 2023;15:e12439. 37192842 10.1002/dad2.12439 PMC10182363

[19] Walter M , Wiltfang J, Vogelgsang J. Pre-analytical sampling and storage conditions of amyloid-beta peptides in venous and capillary blood. J Alzheimers Dis. 2020;78:529-535. 33016918 10.3233/JAD-200777

[20] Ng TKS , Udeh-Momoh C, Lim MA, et al Guidelines for the standardization of pre-analytical variables for salivary biomarker studies in Alzheimer’s disease research: an updated review and consensus of the salivary biomarkers for dementia research working group. Alzheimers Dement 2025;21:e14420. 39737743 10.1002/alz.14420 PMC11848381

[21] Altmann P , Ponleitner M, Rommer PS, et al Seven day pre-analytical stability of serum and plasma neurofilament light chain. Sci Rep. 2021;11:11034. 34040118 10.1038/s41598-021-90639-z PMC8154890

