# Supplementary material for: Storage-dependent variability in Alzheimer disease-related plasma biomarker results using the Fujirebio Lumipulse G1200 platform
Source: J Neuropathol Exp Neurol. 2025 Oct 10;84(12):1143–51. doi: 10.1093/jnen/nlaf115 (PMC12713546; doi:10.1093/jnen/nlaf115)
Supplement: nlaf115_Supplementary_Data [file nlaf115_supplementary_data.docx]

**Supplementary Data.**

**Title:** Storage-Dependent Variability in Alzheimer’s Disease-Related Plasma Biomarker Results Using the Fujirebio Lumipulse G1200 Platform

**Author names and affiliations:** Ibrahim Choucair PhD*^1,2^, Tiffany L Lee BA^2^, Linda J. Van Eldik PhD^2,3^, Jeffrey L. Dage PhD^4,5^, Brian T. Gold PhD^2,3^, Erin L. Abner PhD^2,6^, Gregory A. Jicha MD, PhD^2,7^, Peter T Nelson MD, PhD ^1,2^

^1^Department of Pathology and Laboratory Medicine, University of Kentucky, Lexington KY, USA
^2^Sanders-Brown Center on Aging, University of Kentucky, Lexington KY, USA

^3^Department of Neuroscience, University of Kentucky, Lexington KY, USA

^4^Department of Neurology, Indiana University, Indianapolis IN, USA

^5^Stark Neurosciences Research Institute, Indiana University, Indianapolis IN, USA

^6^Department of Epidemiology & Environmental Health, University of Kentucky, Lexington KY, USA

^7^Department of Neurology, University of Kentucky, Lexington KY, USA

**Corresponding author*:**

Ibrahim Choucair, PhD, NRCC

Department of Pathology and Laboratory Medicine

University of Kentucky College of Medicine

800 Rose Street, HA610

Lexington, KY 40536

Tel: 859-218-5813

Ibrahim.choucair@uky.edu

**Present/permanent address:** Department of Pathology and Laboratory Medicine, University of Kentucky, Lexington, KY, USA

**Supplementary Table 1. Stability study raw data**

| **Storage** | **Time** | **Sample** | **Aβ40 (pg/mL)** | **Aβ42 (pg/mL)** | **pTau181 (pg/mL)** | **pTau217 (pg/mL)** | **NfL (pg/mL)** | **GFAP (pg/mL)** |
| --- | --- | --- | --- | --- | --- | --- | --- | --- |
| Room Temperature | 0 min | A | 290.10 | 32.91 | 0.35 | 0.238 | 3.81 |  |
|  |  | B | 272.88 | 28.74 | 0.51 | 0.106 | 5.55 |  |
|  |  | C | 232.28 | 25.54 | 2.60 | 0.286 | 4.53 | 4.0 |
|  |  | D | 295.40 | 28.84 | 0.53 | 0.103 | 11.85 | 15.3 |
|  | 0.5 hours | A | 297.11 | 32.57 | 0.33 | 0.213 | 4.46 |  |
|  |  | B | 290.74 | 29.22 | 0.50 | 0.100 | 6.05 |  |
|  |  | D | 294.70 | 30.13 | 0.55 | 0.082 | 14.05 | 14.1 |
|  | 1 hour | A | 303.28 | 33.56 | 0.36 | 0.255 | 2.99 |  |
|  |  | B | 280.66 | 25.92 | 0.49 | 0.093 | 6.74 |  |
|  |  | C | 228.37 | 25.67 | 2.73 | 0.332 | 5.41 | 4.0 |
|  |  | D | 267.96 | 28.92 | 0.53 | 0.110 | 14.25 | 15.0 |
|  | 2 hours | A | 284.68 | 32.45 | 0.44 | 0.299 | 3.56 |  |
|  |  | B | 282.52 | 28.54 | 0.54 | 0.099 | 7.95 |  |
|  |  | C | 241.56 | 27.85 | 2.88 | 0.304 | 5.35 | 4.0 |
|  |  | D | 297.84 | 32.27 | 0.60 | 0.100 | 14.15 | 15.4 |
|  | 3 hours | A | 293.08 | 34.3 | 0.50 | 0.318 | 3.60 |  |
|  |  | B | 289.62 | 29.68 | 0.58 | 0.106 | 7.45 |  |
|  |  | C | 239.67 | 25.51 | 2.90 | 0.329 | 5.85 | 4.0 |
|  |  | D | 302.45 | 30.72 | 0.61 | 0.100 | 15.39 | 14.8 |
|  | 4 hours | A | 282.18 | 32.23 | 0.64 | 0.313 | 4.44 |  |
|  |  | B | 282.04 | 29.36 | 0.62 | 0.115 | 7.58 |  |
|  |  | C | 237.57 | 26.50 | 3.03 | 0.330 | 6.29 | 4.0 |
|  |  | D | 297.15 | 31.90 | 0.62 | 0.099 | 14.62 | 14.7 |
| 4 degrees | 1 day | A | 279.84 | 34.07 | 0.61 | 0.191 | 4.31 |  |
|  |  | B | 278.44 | 30.02 | 0.76 | 0.170 | 7.95 |  |
|  |  | C | 223.89 | 18.89 | 2.61 | 0.320 | 6.14 | 4.0 |
|  |  | D | 236.97 | 23.62 | 0.60 | 0.081 | 13.01 | 15.9 |
|  | 5 days | A | 260.12 | 31.55 | 0.39 | 0.082 | 3.18 |  |
|  |  | B | 219.17 | 26.67 | 0.53 | 0.100 | 6.89 |  |
|  | 7 days | C | 158.24 | 15.94 | 2.98 | 0.344 | 8.11 | 4.0 |
|  |  | D | 285.37 | 20.95 | 0.58 | 0.067 | 9.90 | 15.3 |
|  | 10 days | A | 200.85 | 23.14 | 0.64 | 0.054 | 6.31 |  |
|  |  | B | 152.90 | 17.35 | 0.63 | 0.039 | 10.74 |  |
|  |  | C | 132.33 | 12.76 | 2.50 | 0.284 | 5.74 | 4.0 |
|  |  | D | 168.44 | 17.24 | 0.51 | 0.057 | 9.22 | 13.7 |
| -20 degrees | 1 week | A | 288.16 | 29.67 | 2.01 | 0.136 | 4.23 |  |
|  |  | B | 293.08 | 28.26 | 1.75 | 0.103 | 7.18 |  |
|  |  | C | 206.47 | 20.71 | 3.56 | 0.368 | 9.81 | 4.0 |
|  |  | D | 208.94 | 25.42 | 1.46 | 0.096 | 10.26 | 14.5 |
|  | 2 weeks | A | 267.87 | 29.64 | 1.64 | 0.161 | 7.41 |  |
|  |  | B | 252.42 | 26.37 | 1.38 | 0.085 | 11.26 |  |
|  |  | C | 221.31 | 20.41 | 3.10 | 0.344 | 11.33 | 4.0 |
|  |  | D | 275.70 | 25.10 | 1.25 | 0.093 | 9.51 | 12.6 |
|  | 3 weeks | A | 276.78 | 30.59 | 1.44 | 0.189 | 6.85 |  |
|  |  | B | 264.35 | 25.37 | 1.27 | 0.088 | 9.91 |  |
|  |  | C | 221.69 | 21.95 | 3.16 | 0.316 | 11.09 | 20.8 |
|  |  | D | 277.10 | 26.13 | 1.39 | 0.080 | 9.49 | 38.5 |
| -80 degrees | 4 weeks | A | 276.80 | 30.73 | 1.01 | 0.044 | 7.08 |  |
|  |  | B | 277.15 | 29.11 | 0.82 | 0.066 | 10.20 |  |
|  |  | C | 211.13 | 19.31 | 2.95 | 0.323 | 10.99 | 19.4 |
|  |  | D | 281.90 | 24.90 | 0.86 | 0.085 | 12.56 | 37.3 |
|  | 8 weeks | A | 276.12 | 28.27 | 0.83 | 0.062 | 9.49 |  |
|  |  | B | 271.10 | 24.83 | 0.80 | 0.082 | 11.58 |  |

**Supplementary Table 2. 12-Participants stability study demographics and raw data**

|  | **Sample number** | 1 | 2 | 3 | 4 | 5 | 6 |
| --- | --- | --- | --- | --- | --- | --- | --- |
|  | **Cognitive Status** | MCI | MCI | MCI | MCI | MCI | MCI |
|  | **Age** | 74 | 69 | 76 | 92 | 76 | 94 |
|  | **Gender** | Male | Female | Female | Female | Male | Male |
|  | **Race** | White | White | White | Black | White | White |
|  | **MMSE** | 30 | 28 | 25 | 23 | 27 | 28 |
| **Fresh** | **Aβ40 pg/ml** | 392.06 | 384.4 | 342.81 | 381.89 | 374.11 | 377.25 |
| **-80** | **Aβ40 pg/ml** | 374.74 | 391.19 | 308.7 | 356.66 | 365.4 | 387.92 |
| **Fresh** | **Aβ42 pg/ml** | 35.87 | 42.91 | 31.99 | 35.75 | 33.37 | 26.73 |
| **-80** | **Aβ42 pg/ml** | 34.19 | 43.02 | 28.87 | 34.11 | 32.89 | 28.11 |
| **Fresh** | **p181 pg/ml** | 0.94 | 1.56 | 2.28 | 1.13 | 1.79 | 1.59 |
| **-80** | **p181 pg/ml** | 1.58 | 1.78 | 2.55 | 2.52 | 2.43 | 2.47 |
| **Fresh** | **p217 pg/ml** | 0.781 | 0.323 | 0.398 | 1.265 | 0.98 | 0.535 |
| **-80** | **p217 pg/ml** | 0.126 | 0.147 | 0.262 | 0.205 | 0.594 | 0.456 |
| **Fresh** | **NfL pg/ml** | 21.58 | 43.11 | 32.46 | 33.68 | 36.58 | 28.1 |
| **-80** | **NfL pg/ml** | 19.12 | 45.95 | 31.32 | 34.54 | 34.59 | 27.78 |
| **Fresh** | **GFAP pg/ml** | 38.6 | 91.6 | 97 | 183.5 | 47.1 | 133.4 |
| **-80** | **GFAP pg/ml** | 36.4 | 132.1 | 106.4 | 229 | 52.8 | 129.8 |
|  |  |  |  |  |  |  |  |
|  | **Sample number** | 7 | 8 | 9 | 10 | 11 | 12 |
|  | **Cognitive Status** | Normal | Normal | Normal | Normal | Normal | Normal |
|  | **Age** | 80 | 77 | 84 | 73 | 71 | 75 |
|  | **Gender** | Female | Female | Male | Female | Male | Female |
|  | **Race** | White | White | White | White | White | White |
|  | **MMSE** | 30 | 28 | 30 | 30 | 30 | 30 |
| **Fresh** | **Aβ40 pg/ml** | 284.69 | 368.82 | 450.29 | 389.92 | 335.03 | 327.67 |
| **-80** | **Aβ40 pg/ml** | 270.7 | 368.06 | 389.53 | 417.56 | 358.55 | 338.44 |
| **Fresh** | **Aβ42 pg/ml** | 18.25 | 39.74 | 34.42 | 33.2 | 36.29 | 24.88 |
| **-80** | **Aβ42 pg/ml** | 17.98 | 38.82 | 28.06 | 35.69 | 39.7 | 27.27 |
| **Fresh** | **p181 pg/ml** | 1.48 | 1.37 | 2.28 | 3.5 | 1.31 | 1.09 |
| **-80** | **p181 pg/ml** | 1.19 | 1.83 | 3.67 | 4.87 | 1.78 | 2.62 |
| **Fresh** | **p217 pg/ml** | 0.575 | 1.4 | 4.149 | 0.852 | 0.493 | 1.115 |
| **-80** | **p217 pg/ml** | 0.105 | 1.173 | 0.414 | 0.293 | 0.178 | 0.197 |
| **Fresh** | **NfL pg/ml** | 16.95 | 46.11 | 44.56 | 86.27 | 21.81 | 23.11 |
| **-80** | **NfL pg/ml** | 18.73 | 48.2 | 43.66 | 87.88 | 20.92 | 24.27 |
| **Fresh** | **GFAP pg/ml** | 216.2 | 78.2 | 70.8 | 60.5 | 56.2 | 1504.1 |
| **-80** | **GFAP pg/ml** | 34.4 | 112.2 | 106.3 | 87.4 | 70.8 | 58 |

Mild Cognitive Impairment (MCI), Mini-Mental State Examination (MMSE)
